# Yoga and music intervention reduces inattention, hyperactivity/impulsivity, and oppositional defiant disorder in children’s consumer with comorbid ADHD and ODD

**DOI:** 10.3389/fpsyg.2023.1150018

**Published:** 2023-09-20

**Authors:** Xue Luo, Xu Huang, Shuang Lin

**Affiliations:** ^1^School of Business Administration, Southwestern University of Finance and Economics, Chengdu, China; ^2^School of Physical Education, Chengdu Normal University, Chengdu, China; ^3^School of Economics and Management, Shanghai University of Sport, Shanghai, China

**Keywords:** yoga, music intervention, ADHD comorbid with ODD, impulsivity, children, company consumer

## Abstract

**Introduction:**

To analyze the impact of yoga and music intervention on child consumers, we selected 60 eligible child consumers from yoga and music companies.

**Methods:**

This preliminary study used a randomized controlled design to investigate whether a 16-week combined yoga and music intervention improves attention, hyperactivity/impulsivity, and oppositional defiant disorder (ODD) in 60 children with attention-deficit/hyperactivity disorder (ADHD) comorbid with ODD aged 4–6 years. It also preliminarily identified which intervention is best for these children among three types: combined yoga and music, yoga-only, and musiconly interventions. We used both the parent- and teacher-rated MTA SNAP-IV ADHD Rating Scale for data collection.

**Results:**

We found that the combined yoga and music intervention had a positive effect on inattention, hyperactivity/impulsivity, and ODD in children with comorbid ADHD and ODD. The combined yoga and music intervention was the most effective in reducing inattention (in repeated measures ANOVA effect size, 0.9; followed by the yoga- and the music-only interventions, respectively), hyperactivity/impulsivity (effect size, 0.92), and ODD behaviors (effect size, 0.93) in children with comorbid ADHD and ODD. Thus, the combined yoga and music intervention was the most effective and had a more comprehensive effect on children with combined ADHD and ODD compared with the two other interventions (i.e., yoga- and music-only interventions).

**Discussion:**

Our findings provide preliminary evidence for the use of combined yoga and music interventions on a daily basis as a safe and effective adjunctive treatment for children with comorbid ADHD and ODD. The children in the article refer to child consumers of yoga and music companies.

## 1. Introduction

Attention-deficit hyperactivity disorder (ADHD) is a common developmental problem in children, which manifests mainly through the symptoms of attention deficit, hyperactivity, and impulsivity (Beart and Lessing, 2013; Gunaseelan et al., 2021), seriously affecting children’s school and daily lives. Oppositional defiant disorder (ODD) is a group of disorders typically characterized by positional, defiant, and hostile behaviors (and some other forms of behavior) in response to authority figures (Lodber et al., 2000), and the two typical characteristics of children with ODD are irritability and defiance (Leadbeater and Ames, 2017). ODD is the most common comorbidity of ADHD, with a rate of more than 50% (Zou, 2005), and people with comorbid ADHD and ODD are more prone to aggression and externalizing behavioral problems than those with only ADHD (Sun et al., 2001). This study focuses on the case of children with comorbid ADHD and ODD.

In China and in the world, the prevalence of children with ADHD is 5.6% (Li et al., 2018) and 8–10% (Danielson et al., 2018; Jin and Fu, 2022), respectively. Researchers also demonstrate that children with ADHD are 5–10 times more at risk of developing delinquency, antisocial personality disorder, and substance abuse than typically developing children (Chang et al., 2015), causing a huge impact on their families, schools, and society (Bledsoe et al., 2010).

Barkley (1997a,b) constructed a unified theoretical model of ADHD based on theories of the neuropsychological function of the prefrontal lobe, arguing that behavioral inhibition is the central deficit of people with ADHD. It also posits that the three core symptoms of ADHD, namely attention deficit, hyperactivity, and impulsivity, can all be explained by different types of behavioral inhibition (Barkley, 1997a,b). Regarding attention deficit and hyperactivity, they manifest mainly through inattentiveness, incompleteness, emotional impulsivity, and indiscriminate hyperactivity (Yu, 2019), and the thickness of the cerebral cortex can lead to the development of attention deficit and hyperactivity in children (Liang, 2003).

Currently, the main treatment routes for ADHD are pharmacological and psychotherapy. The largest advantage of pharmacological therapy is its fast effects (Valero and Cebolla, 2017), but the side effects of drugs on children, such as insomnia, headache, and drug dependence, cannot be ignored (Meppelink et al., 2016). The long-term prognosis of various approaches to psychotherapy is poor (Wang and Yuan, 2006), and it is not effective in treating mood disorders in children with ADHD (Fang, 2006). Therefore, concomitantly ensuring that treatments for children with comorbid ADHD and ODD are effective and have minimal side effects is an urgent problem that has yet to be solved.

Academicians have indicated that a lack of exercise leads to delayed prefrontal brain development (Hoogman et al., 2019). Mehren et al. (2019) further report that exercise can improve symptoms of inattention, impulsivity, and executive functioning, Montalva-Valenzuela et al. (2022) describe that it can improve cognitive functioning in children, and Welsch et al. (2021) demonstrate that physical activity interventions are valuable complementary treatments for children with ADHD.

In recent years, yoga, which is a form of exercise activity, has been introduced as a potential treatment for children with ADHD (Harrison et al., 2004 Peck et al., 2005 Sheela Joice et al., 2018). Regarding the major scientific evidence about yoga, first, it has been shown to interfere with one of the core symptoms of ADHD, attention deficit, through promoting breathing and meditation (Li et al., 2019), and various scholars corroborate this effect through studies on the impact of yoga-only interventions on children with ADHD. In school settings, yoga therapy is considered a viable intervention to effectively alleviate symptoms in children with ADHD (Steiner et al., 2013). Unlike normal physical activity interventions, yoga practice improves performance on attentional tasks through meditation and breathing, and regularly practicing meditation was associated with visual attention processing and improved sustained attention skills (Hodgins and Adair, 2010). Furthermore, yoga practice is an alternative therapeutic approach that can play an important role in reducing distractions and improving attention (Sheela Joice et al., 2018). Thus, yoga interventions have the potential to improve attention deficits among children with ADHD.

Second, yoga may improve hyperactivity/impulsivity in children with ADHD, and related interventions are described as being promising for tackling behaviors and emotions of children with ADHD (Peck et al., 2005). Furthermore, yoga can reduce symptoms of ADHD and improve cognitive performance in school-aged children (Chaya et al., 2012), as well as reduce inattention and hyperactivity in kindergarten children (Harrison et al., 2004). Therefore, there is detailed empirical evidence and a great deal of focus has been placed on the effects of yoga-only interventions on attention, hyperactivity, and impulsivity in children with ADHD. At the same time, there is a lack of examinations of the effects of interventions combining yoga with other alternative therapeutic approaches, such as music therapy, on children with ADHD—and there is a special paucity of related studies in China. Additionally, regarding children with comorbid ADHD and ODD, only a small number of studies have reported that yoga can improve related symptoms (Jensen and Kenny, 2004; Alam, 2022).

Regarding music therapy, the literature about its effects on the symptoms of children with ADHD is rather small. Among the few researchers that have explored the topic, some describe that the effects are long-lasting and others that these effects are short-lasting and warrant more in-depth study. Particularly, Pratt et al. (1995) demonstrated that background music reduced hyperactive behavior in individuals with ADHD, while Jackson (2003) showcased that music can improve cognitive processes and sensory integration in children with ADHD, as well as that music therapists address these children’s symptoms from multidisciplinary perspectives. Further, Kasuya-Ueba et al. (2020) concluded that music therapy has only a short-term effect on attentional control in children. Despite these reports on the positive effects of music interventions on children with ADHD, they also encompass limitations, which are mostly related to their focus: it was placed more on the effects of music interventions on attention and hyperactivity/impulsivity in ADHD and less on the effects on children with ODD. Academicians have also rarely studied the mechanisms of action of combined yoga and music interventions to improve symptoms in children with ADHD.

Thus, to address the significant aforementioned gaps in the current literature, this study aimed to investigate whether a combined yoga and music intervention improves attention, hyperactivity/impulsivity, and ODD in children with comorbid ADHD and ODD. It also attempted to identify which intervention is best for children with comorbid ADHD and ODD among three intervention types: combined yoga and music, yoga-only, and music-only interventions. The three research questions of this study were as follows: first, does the combined yoga and music intervention improve symptoms in children with comorbid ADHD and ODD and is it a safe and effective daily adjunctive treatment modality for these children? Second, to what extent do the three interventions (i.e., combined yoga and music, yoga-only, and music-only) affect attention, hyperactivity/impulsivity, and ODD in children with comorbid ADHD and ODD, and which intervention has the most effective treatment effect? Third, what are the possible mechanisms of action of the combined yoga and music intervention for children with comorbid ADHD and ODD?

We used both the parent- and teacher-rated MTA SNAP-IV ADHD Rating Scale to rate children with comorbid ADHD and ODD aged 4–6 years before and after the interventions for inattention, hyperactivity/impulsivity, and ODD. Based on the existing, albeit limited, research, we formed two hypotheses:

*Hypothesis 1* (*H1*): The existence of significant differences in the combined intervention of yoga and music on attention, hyperactivity/impulsivity, and ODD in children with comorbid ADHD and ODD will be determined.

*Hypothesis 2* (*H2*): The existence of significant differences in attention, hyperactivity/impulsivity, and ODD between the yoga and music, yoga-only, music-only, and control groups in children with comorbid ADHD and ODD will be determined.

We describe the four major study contributions herein. First, this study fills a research gap regarding the lack of data on the effects of combined yoga and music interventions on children with comorbid ADHD and ODD in China; it also makes a theoretical and practical contribution by expanding the evidence on treatment options for children with comorbid ADHD and ODD. Second, we examined the effects of a combined yoga and music intervention on ODD, implying that the current study was more comprehensive than previous studies that focused solely on core symptoms of ADHD, such as attention deficit and hyperactivity/impulsivity. Third, we compared the effects of three types of intervention (i.e., combined yoga and music, yoga-only, and music-only) on the aforementioned variables in children with comorbid ADHD and ODD, thus providing valuable reference data for the implementation of complementary treatments that can be used daily. Fourth, we preliminarily studied the mechanisms of the impact of combined yoga and music interventions in children with comorbid ADHD and ODD, expanding existing theories and making significant theoretical and practical contributions to the implementation of yoga and music interventions in this population.

## 2. Materials and methods

### 2.1. Research design

This interventional study used a randomized controlled design because randomized controlled trials follow the principles of randomization and control, making them the gold standard for producing reliable clinical evidence (Xu et al., 2020).

**Figure 1:**
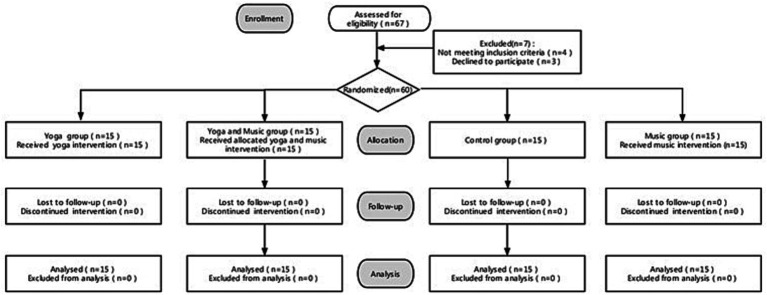
Sampling flow diagram.

Regarding the different interventions, 15 participants in the yoga and music group received a 16-week yoga and music intervention (10 min per yoga intervention and 10 min per music intervention, twice a week); the 15 participants in the control group did not receive any intervention; the 15 children in the yoga-only group received a 16-week yoga intervention (10 min per yoga intervention, twice a week); the 15 participants in the music-only group received a 16-week music intervention (10 min per music intervention, twice a week; Figure 1). Before each intervention session in every group that received an intervention, all participants were asked to take the MTA SNAP-IV ADHD Rating Scale test. After each intervention session in every group that received an intervention, all participants were asked to take the SNAP-IV ADHD Rating Scale again; this served to collect pretest and posttest data. Following the descriptions in the study by Chen et al. (2022), the pretest was conducted 24 h prior to each and every intervention across all groups and the posttest was conducted 24 h after each and every intervention across all groups. These processes were repeated weekly throughout the whole of the intervention period. These processes and data were used to facilitate the detailed documentation of the effects of the yoga and music interventions on children with comorbid ADHD and ODD, but were not included in our analysis. The first pretest measurements were taken 24 h before the start of the study, and the pretest measurements showed no statistically significant differences in Inattention, Hyperactivity/impulsivity, and ODD among the four groups (*p* > 0.05). Regarding the posttest measure of the last intervention session of this study for each group that received an intervention, it was conducted 24 h after the last scheduled intervention (Jarraya et al., 2019). The only data included in the manuscript analyses were the pretest data from before the experiment and the final posttest data measured after the last intervention. Each intervention lasted for 10 min (as aforementioned), and they were performed in the kindergarten of Chengdu Normal University, which was close to the children’s home and convenient for parents to pick up their children after the session. This was also done to ensure adherence, and the children’s attendance rate was 100% for all sessions.

### 2.2. Participants

We used the stratified whole-group random sampling method to divide children’s consumer of yoga company and the music company into three levels: small, middle, and large classes. Three classes were randomly selected from each level to form the sample of this study (Liu et al., 2007). Through this random sampling process, we recruited 67 children’s consumer from music company and the yoga company by distributing flyers in Wenjiang district, Chengdu. The inclusion criteria for participants were as described herein: kindergarten children; having never practiced yoga; diagnosed with comorbid ADHD and ODD by their psychiatrist and confirmed by the school pediatrician (i.e., inattentive, hyperactive/impulsive, ODD, and mixed types were included). Exclusion criteria were as follows: children with a history of neurological disorders; children with a history of brain injury; children with autism spectrum disorders; taking medications other than those used to treat ADHD. Following the methods in prior research (Chou and Huang, 2017), participants who were taking medication were asked to stop taking it at least 24 h before experiment. We assumed a sample size ratio of 1:1:1:1 for the yoga and music, control, yoga-only, and music-only groups, and used SAS 9.4 software to calculate the total sample size required for this study of 60 participants (Figure 1). We assumed a two-sided alpha of 0.05, and we identified effect sizes and conducted power analysis (1 − β) using G*Power, finding that high values of r-squared obtained sufficient power (1 – β) = 0.8 when the sample size of each group was 15 (*n* = 15). Therefore, the power for this study was 0.8.

After screening, 60 children aged 4–6 years were identified as eligible for inclusion, among which 18 were in the small class, 25 in the middle class, and 17 in the large class. All 60 children with comorbid ADHD and ODD (29 females, 31 males) and their legal guardians that partook in this randomized controlled trial volunteered to participate, and all participants and their legal guardians provided written informed consent. All participants and their legal guardians were informed in detail about the design, purpose, and intent of this study, and all study procedures complied with the Declaration of Helsinki and adhered to the basic ethical principles of human research. Furthermore, this study was conducted in compliance with all principles of the Standards for Ethics in Sport and Exercise Science Research (Harriss et al., 2019), and was approved by the Ethics Committee of the College of Physical Education, Chengdu Normal University (approval number: YJYYZY [2021] 02).

Using computer assignment software, the 60 participants were randomly divided into a yoga and music (*n* = 15), control (*n* = 15), yoga-only (*n* = 15), and music-only groups (*n* = 15).

### 2.3. Materials

To assess levels of inattention, hyperactivity/impulsivity, and ODD, we used the MTA SNAP-IV ADHD Rating Scale (see the Appendix). We chose this tool because it showed proper reliability and validity for measuring attention and hyperactivity in children (Lakes et al., 2012; Saxena et al., 2020). Furthermore, the Chinese version of the tool has good psychometric properties, is reliable and valid for assessing ADHD symptoms in Chinese children (Lai et al., 2013), has a high sensitivity in predicting ADHD diagnosis, and is useful for research (Burton et al., 2018).

The MTA SNAP-IV ADHD Rating Scale (Swanson et al., 2001) includes 18 ADHD and 8 ODD symptoms as specified in the Diagnostic and Statistical Manual of Mental Disorders, Fourth Edition (DSM-IV) and International Classification of Diseases 10th Revision (ICD-10) classifications of mental and behavioral disorders. Each symptom is scored by assessing its severity on a 4-point scale (0 = not at all, 1 = somewhat, 2 = about the same, 3 = very much). The items in the DSM-IV Attention Deficit Hyperactivity Disorder criteria contain three subsets of symptoms common in children with ADHD, as follows: inattention (items 1–9), hyperactivity/impulsivity (items 10–18), and ODD (items 19–26). The MTA SNAP-IV ADHD Rating Scale subscale scores (mean scores for each of the inattention, hyperactivity/impulsivity, and ODD subscales) are calculated by adding the scores of the subscales’ items and dividing by the number of items in the subscale (Swanson et al., 2001). The MTA SNAP-IV ADHD Rating Scale includes separate scores for parents and teachers, and the scores (inattentive, hyperactive/impulsive, and ODD) for each child in this study were averaged based on the parent and teacher scores. The parents and the teachers responded to their respective versions of the MTA SNAP-IV ADHD Rating Scale under the guidance and supervision of psychiatrists. Both parents and teachers were required to objectively evaluate the child’s three ADHD symptom subsets, and the parents that responded to the scale were those who had the most contact with the child.

The Cronbach’s alpha coefficient of the Chinese version of the MTA SNAP-IV ADHD Rating Scale and its three subscales of inattention, hyperactivity/impulsivity, and ODD were 0.95, 0.90, 0.89, and 0.88, respectively. In a past study by Zhou et al. (2013), the intraclass correlation coefficient of the retest reliability for the total scale and its subscales were 0.68, 0.75, 0.76, and 0.24, respectively. The authors described that the retest reliability (Intraclass Correlation Coefficient, ICC) of the ODD subscale in the Chinese version of the MTA SNAP-IV ADHD Rating Scale was 0.24. Thus, the Chinese version of the MTA SNAP-IV ADHD Rating Scale has good reliability.

The correlation coefficients between the Chinese version of MTA SNAP-IV ADHD Rating Scale and the Parent Symptom Questionnaire’s hyperactivity, impulsivity, and conduct factors ranged from 0.39 to 0.72 (*p* < 0.01; Zhou et al., 2013). The correlation coefficients between the attention and aggression factors of the MTA SNAP-IV ADHD Rating Scale and Child Behavior Checklist ranged from 0.30 to 0.74 (*p* < 0.01; Zhang et al., 2016). In the studies by Zhou et al. (2013) and Zhang et al. (2016), the Chinese version of the MTA SNAP-IV ADHD Rating Scale correlated with the Chinese version of the Achenbach Child Behavior Checklist (CBCL) for the attention problems, violations, and aggressive behavior factors, with the coefficients ranging from 0.3 to 0.74. This shows that the Chinese version of the MTA SNAP-IV ADHD Rating Scale has good validity.

The Chinese version of the MTA SNAP-IV ADHD Rating Scale has good psychometric properties and is suitable for assessing changes in ADHD symptoms in Chinese children (Zhou et al., 2013; Zhang et al., 2016). The Chinese version of the MTA SNAP-IV ADHD Rating Scale has good sensitivity and high convergent validity for assessing symptoms of ADHD comorbid with ODD.

### 2.4. Procedure

In each intervention, the yoga and music group began with a 10-min yoga intervention; after a 1-h interval, a 10-min music listening intervention was performed. This occurred twice a week for 4 months (16 weeks).

The yoga intervention (Table 1) includes 5 min of meditation and 5 min of yoga poses led by an experienced yoga instructor. This instructor also defined the program’s content, and to ensure that the program was safe and appropriate for children, the contents defined by the instructor were checked by a panel of yoga experts with the power to vet any content that was potentially unsafe or inappropriate. In each yoga class, the instructor selected 2–3 yoga asanas (presented in Table 1) to teach the children.

**Table 1:** Specific yoga poses.

	Position	Yoga poses	Time
I	Breathing and Meditation	Pranayama (breathing and meditation)	5 min
II Postures (asanas)	Standing postures	Mountain pose Chair pose Rag doll pose Tree pose	5 min
	Sitting postures	Cow face pose Bound-angle pose Head-to-knee pose Child’s pose	
	Supine postures	Bridge pose Big toe pose Happy baby pose Corpse pose	

The music intervention was performed by playing music that the children liked in a quiet classroom at the kindergarten of Chengdu Normal University, wherein participants chose their favorite track among some common children’s songs and listened to it for 10 min. Examples of the Chinese children’s songs used are “Little Star,” “Red Dragonfly,” “Jingle Bells,” and “Wahaha.” The rationale behind the choice of these tracks is described in the study by Sholeh and Supena (2021), which proposes that songs for children can be used as a medium to treat and overcome anxiety and help children with ADHD to better control themselves. To ensure the successful completion of the music intervention, prior to intervention onset, the teacher informed the children that they would get a reward (e.g., a small red flower) if they listened to a track of their choice for 10 min.

### 2.5. Data analysis

Data analysis was performed using SPSS, version 26.0. First, we checked the data for normality, variance chi-square, and the presence of outliers. We found no outliers, no variable showed a highly non-normal distribution, and based on a cut-off for the Shapiro–Wilk test of >0.05, all data were normally distributed (Table 2). Second, we used descriptive statistics to analyze participants’ sociodemographic characteristics. Third, we used independent t-tests to compare the difference in scores between group pairs by sociodemographic characteristics. Fourth, repeated measures analysis of variance was employed to analyze the effects of the interventions on children with comorbid ADHD and ODD. Statistical significance was set at a *p* < 0.05.

**Table 2:** Descriptive statistics of the data for the key study variables.

Variables	Time	M	SD	Skewness	Kurtosis	Shapiro–Wilk Value of *p*
Inattention	Pretest	2.40	0.21	0.002	−1.165	>0.05
	Posttest	2.00	0.32	−0.211	−0.386	>0.05
Hyperactivity/impulsivity	Pretest	2.39	0.23	−0.037	−1.064	>0.05
	Posttest	1.99	0.34	0.195	−1.316	>0.05
ODD	Pretest	2.34	0.18	−0.042	−0.919	>0.05
	Posttest	1.97	0.29	0.293	−1.182	>0.05

ODD, oppositional defiant disorder.

## 3. Results

All 60 participating children lived in the Wenjiang District of Chengdu and were from families of middle socioeconomic status. For the total sample, the mean age, height, and weight was 4.98 years (standard deviation [SD], 0.77 years), 109.33 cm (SD, 6.67 cm), and 20.9 kg (SD, 3.02 kg), respectively. Regarding mean age and height, the group with the highest value was the control group (age: 5.07 years, SD, 0.88 years; height: 110.93 cm, SD, 7.92 cm). Regarding mean weight, the group with the highest value was the yoga group (21.67 kg, SD, 2.13). Table 3 shows that there were no statistically significant differences in age, height, and weight between any groups (*p* > 0.05).

**Table 3:** Participants’ characteristics.

Variables	Yoga and music group (*n* = 15) Mean (±SD)	Control group (*n* = 15) Mean (±SD)	Yoga group (*n* = 15) Mean (±SD)	Music group (*n* = 15) Mean (±SD)	Total (*N* = 60) Mean (±SD)
Age (years)	4.93 ± 0.80	5.07 ± 0.88	4.94 ± 0.70	5.00 ± 0.76	4.98 ± 0.77
Height (cm)	109.13 ± 7.17	110.93 ± 7.92	108.87 ± 5.93	108.40 ± 5.85	109.33 ± 6.67
Weight (kg)	20.00 ± 3.44	20.40 ± 3.72	21.67 ± 2.13	21.53 ± 2.45	20.9 ± 3.02

In our study, the internal consistency reliability Cronbach’s alpha coefficient of the Chinese version of the MTA SNAP-IV ADHD Rating Scale was 0. 94, and the Cronbach’s alpha coefficients of the three subscales of inattention, hyperactivity/impulsivity, and ODD were 0.89, 0.88, and 0.87, indicating good reliability of our measure. We performed validated factor analysis of the Scale using Amos software, with RMSEA <0. 05, $X^{2}/df<2$, CFI > 0.9, factor loadings >0.7 for all 26 questions, AVE > 0.5, and significant correlations between the 3 dimensions of inattention, hyperactivity/impulsivity, and ODD (*p* < 0. 05). These results indicate that our measure has good validity. This shows that the scale we used has good psychometric properties.

**Figure 2:**
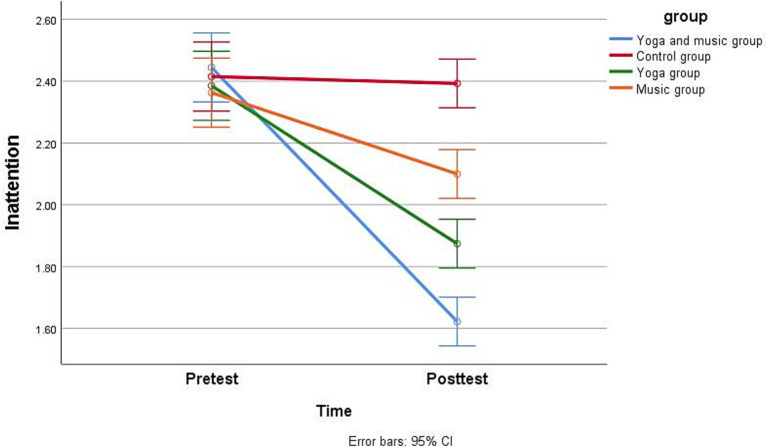
Participants’ pretest and posttest scores for inattention by group.

**Figure 3:**
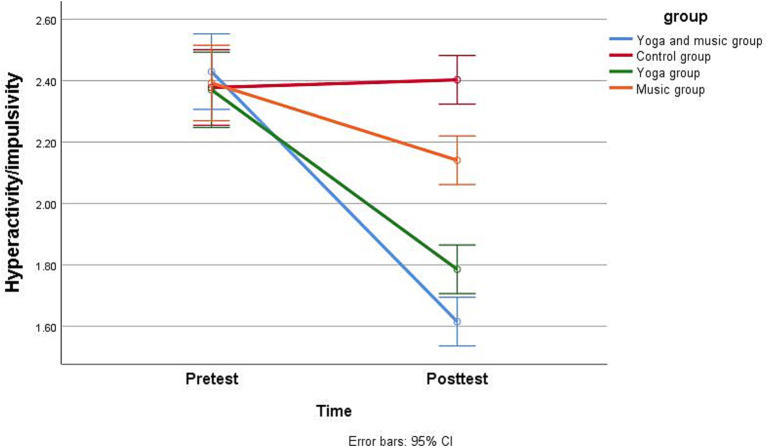
Participants’ pretest and posttest scores for hyperactivity/impulsivity by group.

**Figure 4:**
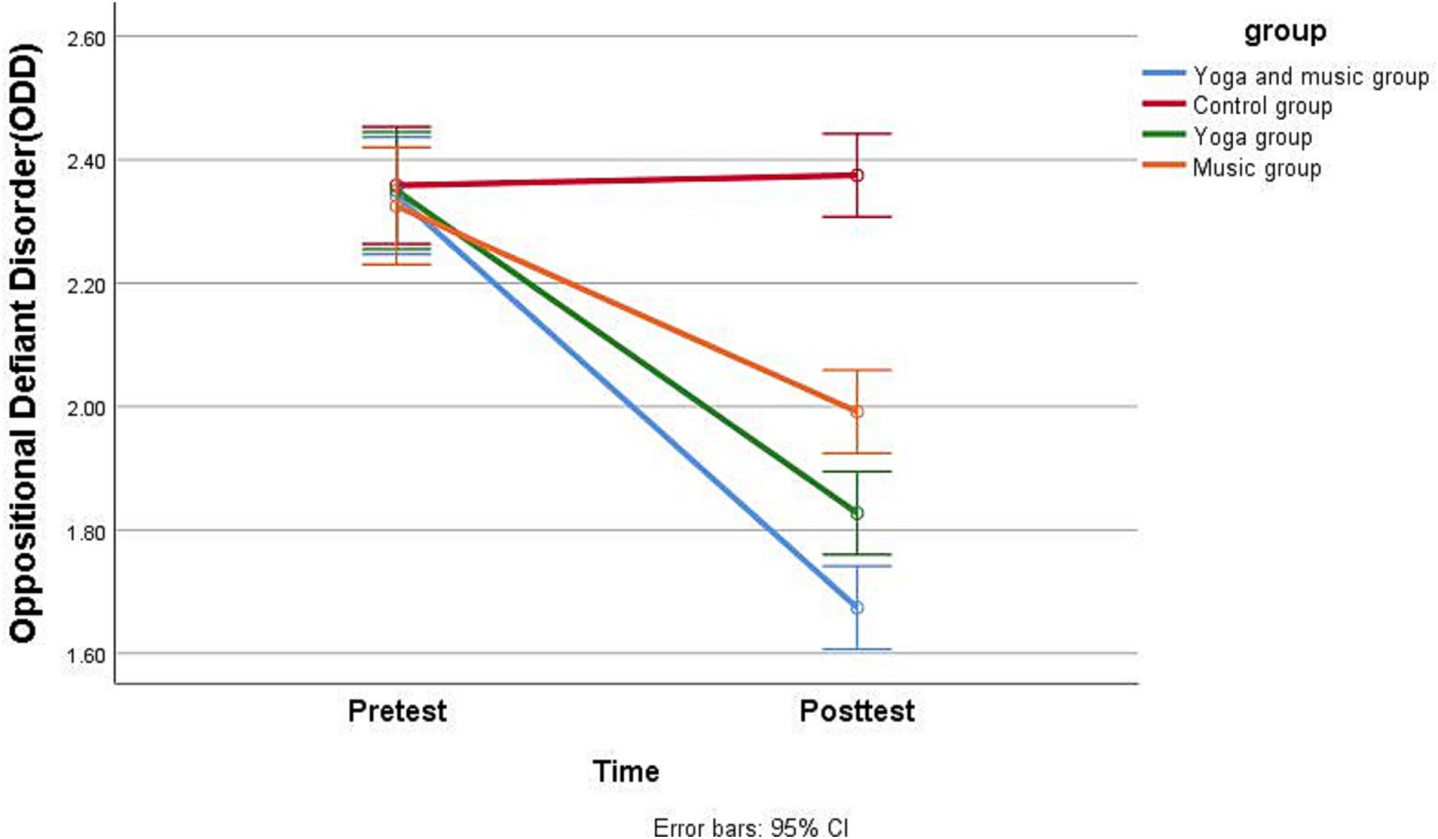
Participants’ pretest and posttest scores for oppositional defiant disorder by group.

Regarding *H1*, as shown in Tables 4, 5, the repeated measures analysis of variance for comparing pretest and posttest scores revealed that the combined yoga and music intervention significantly reduced inattention (Figure 2), hyperactivity/impulsivity (Figure 3), and ODD scores (Figure 4) in children with comorbid ADHD and ODD (*p* < 0.05). The results suggest that the combined yoga and music intervention can help children with comorbid ADHD and ODD focus their attention and reduce hyperactivity/impulsivity and ODD behaviors.

**Table 4:** Within group and between group comparisons of ADHD comorbid with ODD before and after interventions.

Variables	Yoga and music group		Effect size	Control group (no intervention)		Effect size
	Pretest	Posttest		Pretest	Posttest	
Inattention	2.44 ± 0.19	1.62 ± 0.21	0.90	2.41 ± 0.22	2.39 ± 0.12	0.06
Hyperactivity/impulsivity	2.43 ± 0.22	1.61 ± 0.10	0.92	2.38 ± 0.23	2.40 ± 0.14	−0.05
ODD	2.34 ± 0.16	1.67 ± 0.09	0.93	2.36 ± 0.17	2.37 ± 0.07	−0.04
**Variables**	**Yoga group**		**Effect size**	**Music group**		**Effect size**
	**Pretest**	**Posttest**		**Pretest**	**Posttest**	
Inattention	2.39 ± 0.24	1.87 ± 0.13	0.80	2.36 ± 0.21	2.10 ± 0.13	0.60
Hyperactivity/impulsivity	2.37 ± 0.25	1.79 ± 0.16	0.81	2.39 ± 0.25	2.14 ± 0.20	0.48
ODD	2.35 ± 0.18	1.83 ± 0.17	0.83	2.33 ± 0.22	1.99 ± 0.16	0.66

ODD, oppositional defiant disorder.

**Table 5:** Results of the analysis of variance.

Variables	Source	*F* (*df*)	Value of *p*	Partial *η*2	Observed power
Inattention	Time point	451.29 (1)	<0.05	0.89	1.0
	Time point × group	80.81 (3)	<0.05	0.81	1.0
Hyperactivity/impulsivity	Time point	422.48 (1) 422.48	<0.05	0.88	1.0
	Time point × group	87.03 (3)	<0.05	0.82	1.0
ODD	Time point	615.78 (1)	<0.05	0.92	1.0
	Time point × group	94.79 (3)	<0.05	0.84	1.0

For *H2*, as shown in Tables 4, 5, the repeated measures analysis of variance revealed that the combined yoga and music intervention had the best adjunctive treatment effect for children with comorbid ADHD and ODD. Regarding inattention, the intervention effect sizes were 0.9, 0.8, 0.6, and 0.06 for the yoga and music, yoga-only, music-only, and the control groups, respectively, indicating that the combined yoga and music intervention was the most effective in reducing inattentive behavior in children with comorbid ADHD and ODD. Regarding hyperactivity/impulsivity, the yoga and music group showed the largest effect size (0.92), while that for the yoga-only group (0.81) was greater than that for the music-only group (0.48), and the intervention effect size of the control group was −0.05; thus, the combined yoga and music intervention was the most effective in reducing hyperactivity/impulsivity behaviors in children with comorbid ADHD and ODD. Regarding ODD, the intervention effect sizes were 0.93, 0.83, 0.66, and − 0.04 for the yoga and music, yoga-only, music-only, and control groups, indicating that the combined yoga and music intervention was the most effective in reducing ODD behaviors in children with comorbid ADHD and ODD.

To compare whether there were statistically significant differences between the yoga and music, yoga-only, music-only, and control groups, we conducted multiple comparisons of group data using one-way analysis of variance. The posttest data for inattention, hyperactivity/impulsivity, and ODD data in the four groups met the conditions of normality and Chi-squareness. The results of the posttest data showed that there were no significant differences between the yoga and music groups in terms of ODD (*p* = 0.064), while the remaining groups showed significant differences between inattention, hyperactivity/impulsivity, and ODD (*p* < 0.05).

## 4. Discussion

Our study investigated the effects of a combined yoga and music intervention on attention, hyperactivity/impulsivity, and ODD in children with comorbid ADHD and ODD using both the parent- (pretest and posttest) and the teacher-rated (pretest and posttest) MTA SNAP-IV ADHD Rating Scales. We found that the combined yoga and music intervention had a positive effect on attention, hyperactivity/impulsivity, and ODD, supporting *H1*. Furthermore, the combined yoga and music intervention was the most effective and had a more comprehensive effect in reducing inattention (followed by the yoga- and music-only interventions), hyperactivity/impulsivity (followed by the yoga- and music-only interventions), and ODD behaviors, confirming *H2*. We also preliminarily verified that the music intervention enhanced the effect of the yoga intervention, and that the latter helped the former’s effect last longer.

The confirmation of *H1*, while using the parent- and teacher-rated MTA SNAP-IV ADHD Rating Scales, implies that 16 weeks of a combined yoga and music intervention can positively significantly impact children with comorbid ADHD and ODD. Specifically, it can help them to focus their attention and reduce hyperactivity/impulsivity and ODD behaviors. Thus, this combined intervention may be used on a daily basis and as an optional, safe, effective adjunctive therapy for children with comorbid ADHD and ODD. Our study fills a gap in the literature regarding the lack of evidence on the effects of combined yoga and music interventions on ADHD symptoms in Chinese children. Additionally, although researchers thus far have mostly focused their examinations on the effects of yoga- and music-only interventions on attention and hyperactivity in children with ADHD, our study focused additionally on ODD and compared the effects of the combined yoga and music intervention with those of yoga- and music-only interventions—with the findings supporting *H2*. Thus, this study provides more comprehensive research data than previous studies and enriches the evidence on the effects of joint yoga and music interventions on children with comorbid ADHD and ODD, making both theoretical and practical contributions to the field.

Our study shows the positive effect of yoga interventions on attention and hyperactivity/impulsivity in children with comorbid ADHD and ODD, and this is consistent with the evidence in prior research on children with ADHD (Doulou and Drigas, 2022; Fritz and O’Connor, 2022). Yoga practice positively regulates mental states and attention through its breathing and posture activities (Chou and Huang, 2017), and meditation is effective in reducing ADHD symptoms in children (Ramos et al., 2022). Palmer et al. (2013) also described that kindergarten children have more sustained attention after practicing yoga because the prefrontal brain regions involved in sustained attention are activated after exercise. Yoga practice has also been shown to be associated with reduced cortisol and increased dopamine (Pal et al., 2014), and the long-term regular practice of yoga (more than 8 weeks) can significantly enhance inhibitory function and increase dopamine in children with ADHD (Ren et al., 2022).

Considering the paucity of evidence on the effects of yoga interventions on ODD behaviors in children with ADHD, in addition to attention and hyperactivity/impulsivity, we examined the effects of a combined yoga and music intervention on ODD—and as aforementioned, the reduction in this variable was significant. Irritability and defiance are the two typical characteristics of children with ODD (Leadbeater and Ames, 2017), and we believe that because yoga emphasizes one’s control of own body, breath, and consciousness, its regular practice helps children with comorbid ADHD and ODD control their consciousness and emotions, thereafter curbing the experience of negative emotions (e.g., irritability and defiance). Regarding the effects of music interventions, researchers demonstrate that they can release dopamine by stimulating the secretion of hormones associated with feelings of well-being (Salimpoor et al., 2011); this may be important for children with comorbid ADHD and ODD because, as shown by Mariggiò et al. (2021), defects in dopamine are associated with the pathogenesis of ADHD. Additionally, music interventions can improve participants’ self-confidence and social skills (MacGlone et al., 2020), music is a mechanism for cognitive mood change (Chan and Han, 2022), and music can regulate mood (Aalbers et al., 2022). Thus, music interventions help children with comorbid ADHD and ODD to experience positive emotions, and a combined yoga and music intervention can inhibit their ODD behaviors (i.e., improve behavioral problems related to ODD).

As quickly remarked in the prior paragraph, our findings suggest that music therapy has a positive impact on children with comorbid ADHD and ODD. This is corroborated by prior research, which shows that such therapy can enhance attention and reduce hyperactivity in children with ADHD (Kossyvaki and Curran, 2020). Music therapy helps with self-regulation and the control of impulsive behaviors (Saraswati and Mohan, 2021), and music *per se* can stimulate the brain. Other researchers show that music can have positive psychological effects in the treatment of ADHD (Park et al., 2022), as well as that music therapy can enhance the effectiveness of yoga practice and greatly relax the brain after stress (Weerasinghe and Joniton, 2021). When participants in a prior study listened to or played music, their ADHD symptoms were reduced or disappeared completely (Wilde and Welch, 2022). Furthermore, the interaction between musical rhythms can stimulate emotions and relax the mind (Vajpeyee et al., 2022), and music reduces ADHD symptoms by influencing mood, cognition, and behavior (Zemestani et al., 2023).

In summary, the current study shows that combined yoga and music interventions are the most effective and comprehensive (vs. yoga- and music-only interventions) for use in children with comorbid ADHD and ODD, as well as preliminarily verifies that while the music intervention reinforces the effects of the yoga intervention, the latter helps the effects of the first to last longer. The results demonstrate the reasonableness of our hypotheses and the feasibility of this combined intervention in the treatment of children with comorbid ADHD and ODD. We tentatively suggest that the mechanism of action of this approach may be related to the development of the prefrontal lobe of the brain, especially because frontal lobe function impairment is a core deficit in children with ADHD (Castellanos and Tannock, 2002; Xu and Jiang, 2021). Researchers have shown that yoga intervention increases the volume of the prefrontal cortex (Cai and Zhang, 2019; Chen et al., 2022), and listening to music involves several areas of the brain, including cortical areas (Chan and Han, 2022). Specifically, musical intervention strongly activates the ventral tegmental area, insula, nucleus accumbens, and hypothalamus, and the activation of these sites aids the release of dopamine *in vivo* (Gfeller et al., 2000; Shu and Zhang, 2021). This release, in turn, has been shown to increase the volume of the prefrontal cortex and the number of neurotransmitters and receptors in the brain (Yang et al., 2022), thereafter potentially promoting symptom improvements for children with comorbid ADHD and ODD. Therefore, the relationship between yoga and music interventions may be complementary and mutually reinforcing. We hope that, in the future, scholars pay more attention to the combined intervention of yoga and music as an assisted treatment for children with ADHD and ODD; a relevant research effort would be further examining the mechanism of action of the combined intervention of yoga and music, so that more confirmations and explanations can be provided for the assisted treatment of children with comorbid ADHD and ODD with yoga and music.

Our study has high practical value, and the related suggestions are as follows. Our results point out that the combined yoga and music intervention can serve as a daily adjunctive therapy for children with comorbid ADHD and ODD. Accordingly, the government could develop a yoga and music curriculum suitable for children with comorbid ADHD and ODD, organize trainings for teachers and parents on yoga and music-assisted therapy, and conduct related lectures to promote the widespread use of yoga and music therapy. Schools can arrange yoga and music programs according to the characteristics of their children, and use classroom or extracurricular activities to provide these complementary forms of therapy for children with comorbid ADHD and ODD. Parents can arrange parent–child yoga activities and music activities, which may not only ensure the continuity of intervention therapies for ADHD and ODD in the family setting but also enhance the parent–child relationship.

### 4.1. Limitations and future studies

Despite its numerous strengths and novel data on combined yoga and music interventions, this study also has some limitations. First, only one tool, the MTA SNAP-IV ADHD Rating Scale, was used to evaluate the effect of the combined intervention on children with comorbid ADHD and ODD. Future studies could use various scale types, including the Conners Child Behavior Questionnaire and the Piers-Harris Child Self-Awareness Inventory, to evaluate the effects of the joint intervention on the inattention, hyperactivity/impulsivity, and ODD of children with comorbid ADHD and ODD. This will surely provide more confirmatory evidence as to the extent and how the intervention can assist in the treatment of children with comorbid ADHD and ODD.

Second, while we did preliminarily verify *H2* and proposed the potential action mechanism behind the combined yoga and music intervention on children with comorbid ADHD and ODD, researchers could advance this research topic (e.g., by studying other action mechanisms) and check whether their future findings corroborate these preliminary results. We believe that there is still much room for related research, and we expect that academicians can continue to refine our understanding of the mechanisms underlying the influence of combined yoga and music interventions on ADHD and ODD symptoms in children.

Third, upon analyzing our own results, we noticed that a question was begged: what is the effect and mechanism of action of the combined yoga and music intervention on children with different subtypes of ADHD (i.e., attention deficit dominant type, hyperactivity/impulsivity dominant type, and mixed type)? Our current results do not approach this topic, very few scholars have delved into it, and this is a potential focus of our own future research.

Fourth, our study was a preliminary pilot study with a relatively small sample size per group. Researchers could endeavor to expand the sample size of their future studies in order to provide more evidence on the effects of a combined yoga and music intervention on children with comorbid ADHD and ODD. In addition, there is the possibility of reporting bias in the study, that future researchers could make use of more objective measures of inattention in a fully powered trial in order to deliver complementary evidence for this variable.

## 5. Conclusion

This study evaluated the effects of a combined yoga and music intervention on attention, hyperactivity/impulsivity, and ODD in children with comorbid ADHD and ODD using both the parent- and teacher-rated MTA SNAP-IV ADHD Rating Scales. The evidence suggests that the combined yoga and music intervention is a safe and effective complementary treatment for children with comorbid ADHD and ODD and can be used on a daily basis. Particularly, the combined yoga and music intervention was the most effective and had a more comprehensive effect than the yoga- and the music-only interventions. Future research on combined yoga and music interventions for children with comorbid ADHD and ODD could focus on the effects of such interventions for children with different types of ADHD and their mechanisms of action. A greater focus on combined yoga and music interventions as being safe, simple, and effective can provide us with a more comprehensive understanding of the treatment of children with comorbid ADHD and ODD.

The significance and value of this study lies in its examination of the positive effects of a combined yoga and music intervention on inattention, hyperactivity/impulsivity, and ODD in children with comorbid ADHD and ODD, enriching the evidence on complementary treatments for this population. Compared with pharmacological therapy, the combined yoga and music intervention has the advantages of being side-effect free, lower cost, healthier, and more accessible, meaning that our study provides a safe and overall better option for a complementary treatment of children with comorbid ADHD and ODD.

Future research could further explore the mechanisms of action of combined yoga and music in interventions for children with comorbid ADHD and ODD, compare different combined yoga and music intervention protocols, and determine which protocols are most effective in treating children with comorbid ADHD and ODD. An appropriate research question for a future study could be as follows: what are the effects and mechanisms of action of combined yoga and music interventions on different subtypes of children with ADHD (i.e., attention deficit dominant, hyperactive/impulsive dominant, and mixed)? Future studies could also collect data from larger samples and use longitudinal designs to track the long-term benefits of yoga and music for children with comorbid ADHD and ODD across the lifespan, which may allow for a broader generalization of the findings.

## Data availability statement

The original contributions presented in the study are included in the article/supplementary material, further inquiries can be directed to the corresponding author.

## Ethics statement

All participants and their legal guardians provided written informed consent. All participants and their legal guardians were informed in detail about the design, purpose, and intent of this study. The study was approved by the Ethics Committee of the College of Physical Education, Chengdu Normal University.

## Author contributions

XL designed the study and wrote the manuscript. XH revised the manuscript. SL revised the manuscript. All authors contributed to the article and approved the submitted version.

## Appendix

The MTA SNAP-IV Rating Scale for ADHD (SWAN) (Swanson et al., 2001).
